# Supplementary material for: Future phytoplankton diversity in a changing climate
Source: Nat Commun. 2021 Sep 10;12:5372. doi: 10.1038/s41467-021-25699-w (PMC8433162; doi:10.1038/s41467-021-25699-w)
Supplement: Supplementary file 1 — Supplementary Information [file 41467_2021_25699_MOESM1_ESM.pdf]

Supplementary Information

for

Future phytoplankton diversity in a changing climate

Stephanie A. Henson, B. B. Cael, Stephanie R. Allen, and Stephanie Dutkiewicz

This file contains 5 Supplementary Figures.

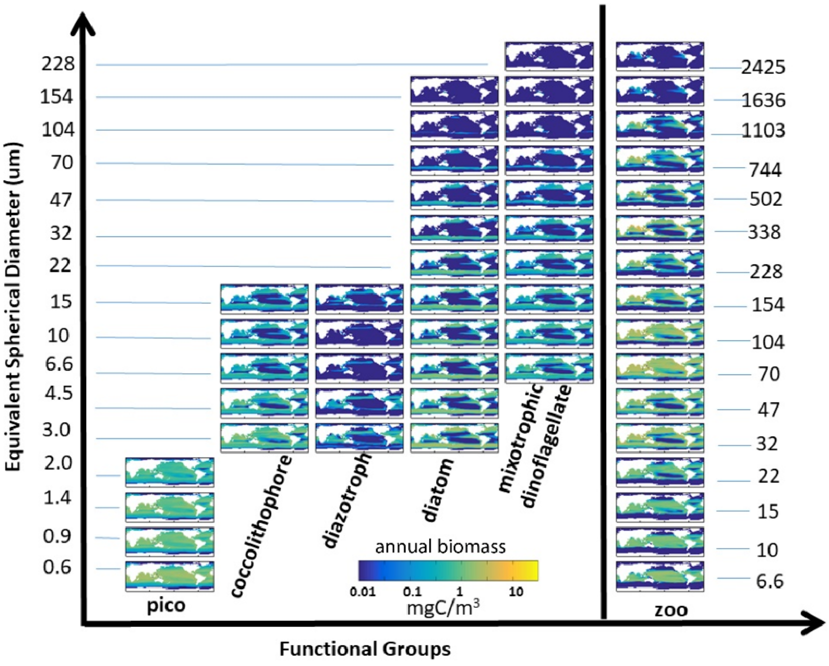

Supplementary Figure 1: Annual biomass (mg C m^{-3}) over the upper 50 m for each of the 51 plankton types for the present day. Columns denote different functional groups, and rows indicate the size (equivalent spherical diameter, µm). Note that the zooplankton have a different size range (right y-axis).

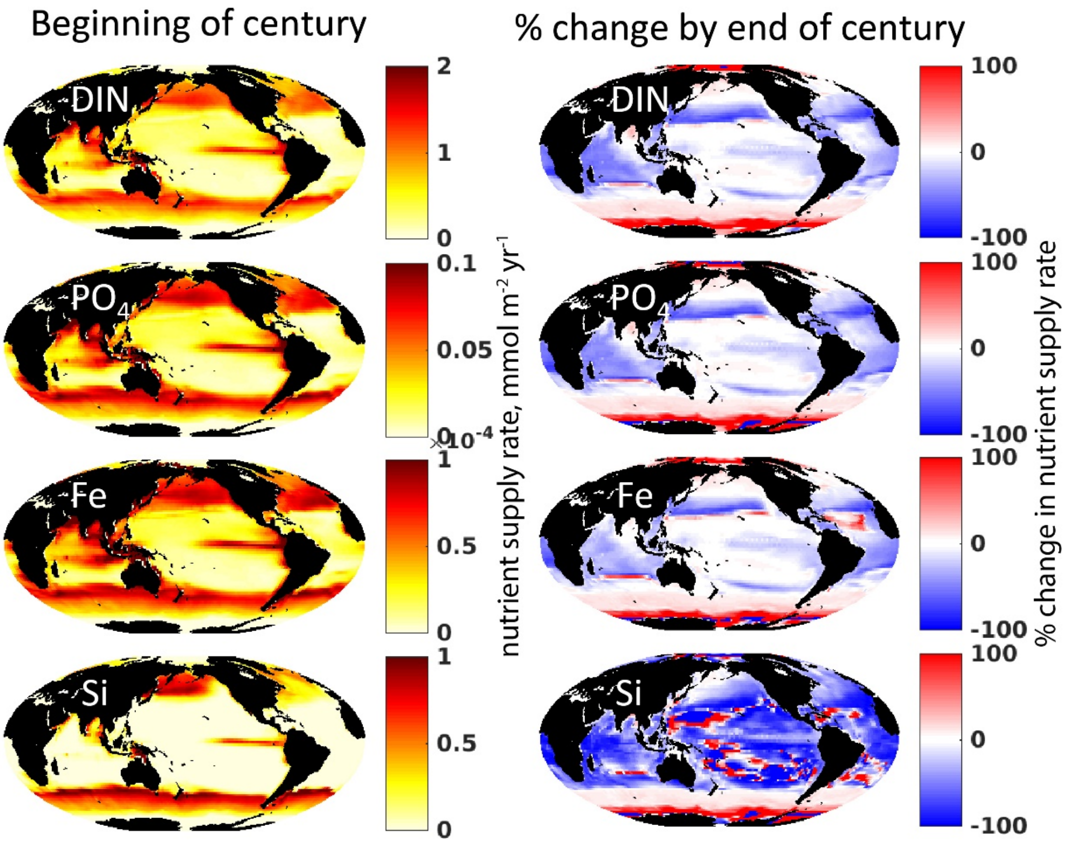

Supplementary Figure 2: (left) Nutrient supply rates into the top 40m of the model ocean from 2005-2024 (mmol m^{-2} $year^{-1}$). Top to bottom: supply rate of dissolved inorganic nitrogen (DIN), phosphate (PO_4), dissolved bioavailable iron (Fe) and silicic acid (Si). (right) Percentage change in the supply rates by the end of the century (2081-2100). Blue indicates a reduction in nutrient supply rate.

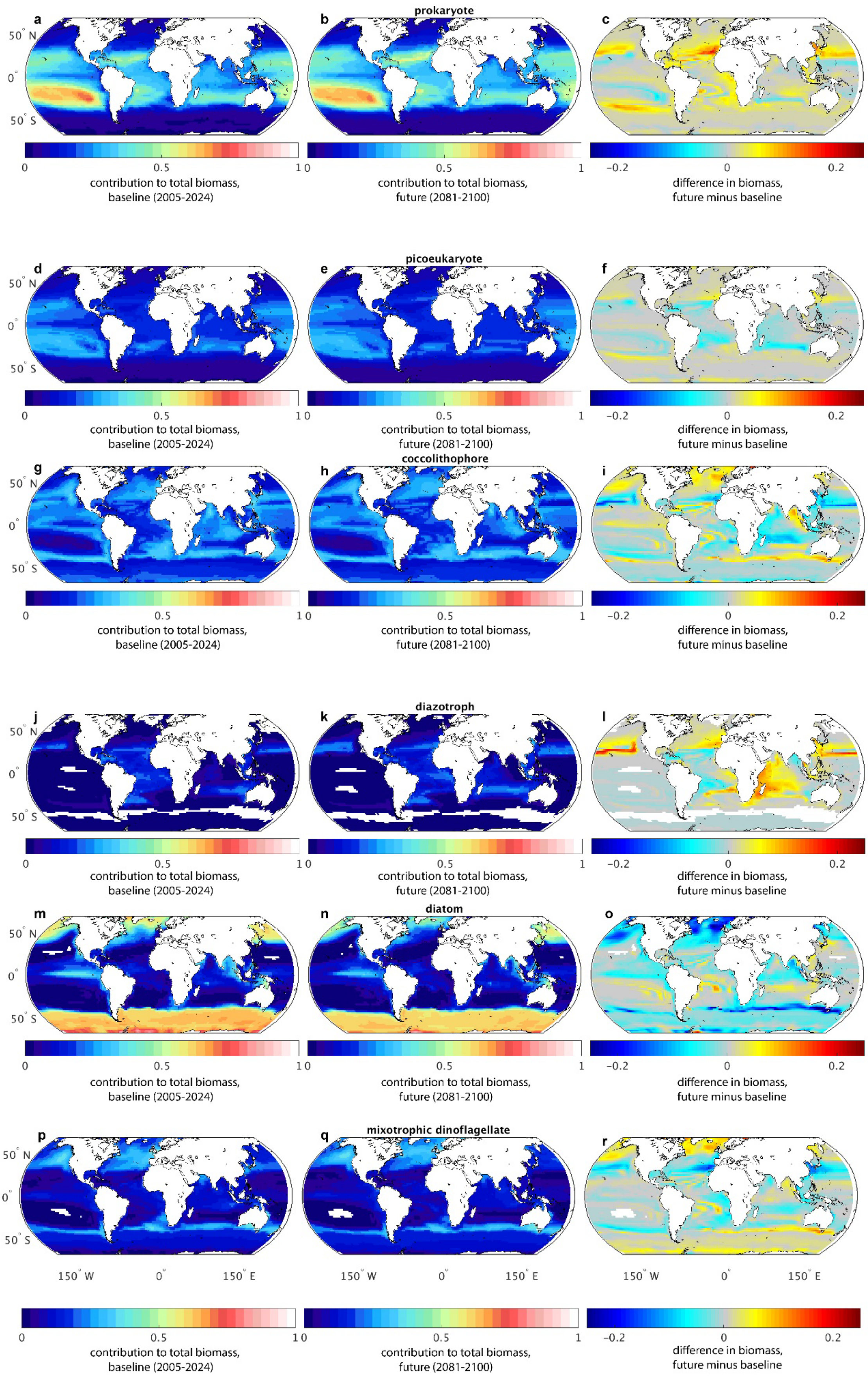
prokaryote
picoeukaryote
coccolithophore
diazotroph
diatom
mixotrophic dinoflagellate
a
b
c
d
e
f
g
h
i
j
k
l
m
n
o
p
q
r
50° N
0°
50° S
150° W
0°
150° E
0
0.5
1
-0.2
0.2
contribution to total biomass, baseline (2005-2024)
contribution to total biomass, future (2081-2100)
difference in biomass, future minus baseline

Supplementary Figure 3: Fractional contribution of 6 phytoplankton types to total biomass: mean of baseline period (2005-2024) in left column, mean of end of century period (2081-2100) in centre column, and difference between the end of century and baseline periods in right column. a-c) prokaryotes, d-f) picoplankton, g-i) coccolithophore, j-l) diazotrophs, m-o) diatoms and p-r) mixotrophic dinoflagellates.

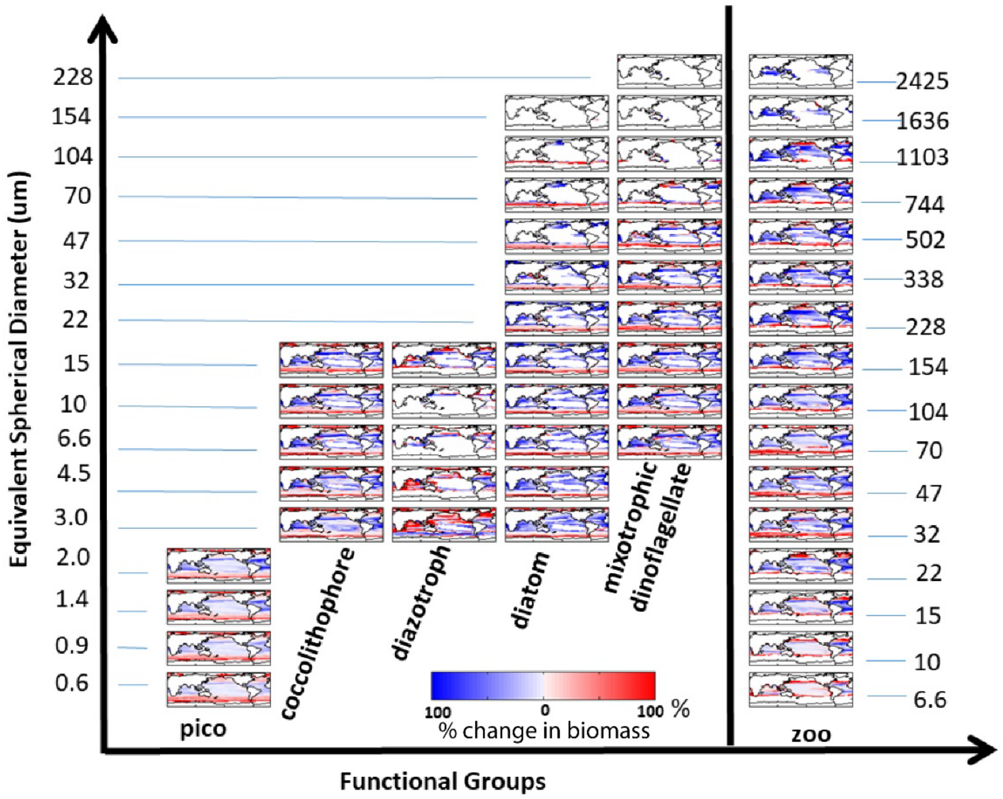

Supplementary Figure 4: Fractional change in biomass over the upper 50 m for each of the 51 plankton types between the means of 2005-2024 and 2081-2100. Columns denote different functional groups, and rows indicate the size (equivalent spherical diameter, μm). Note that the zooplankton have a different size range (right y-axis).

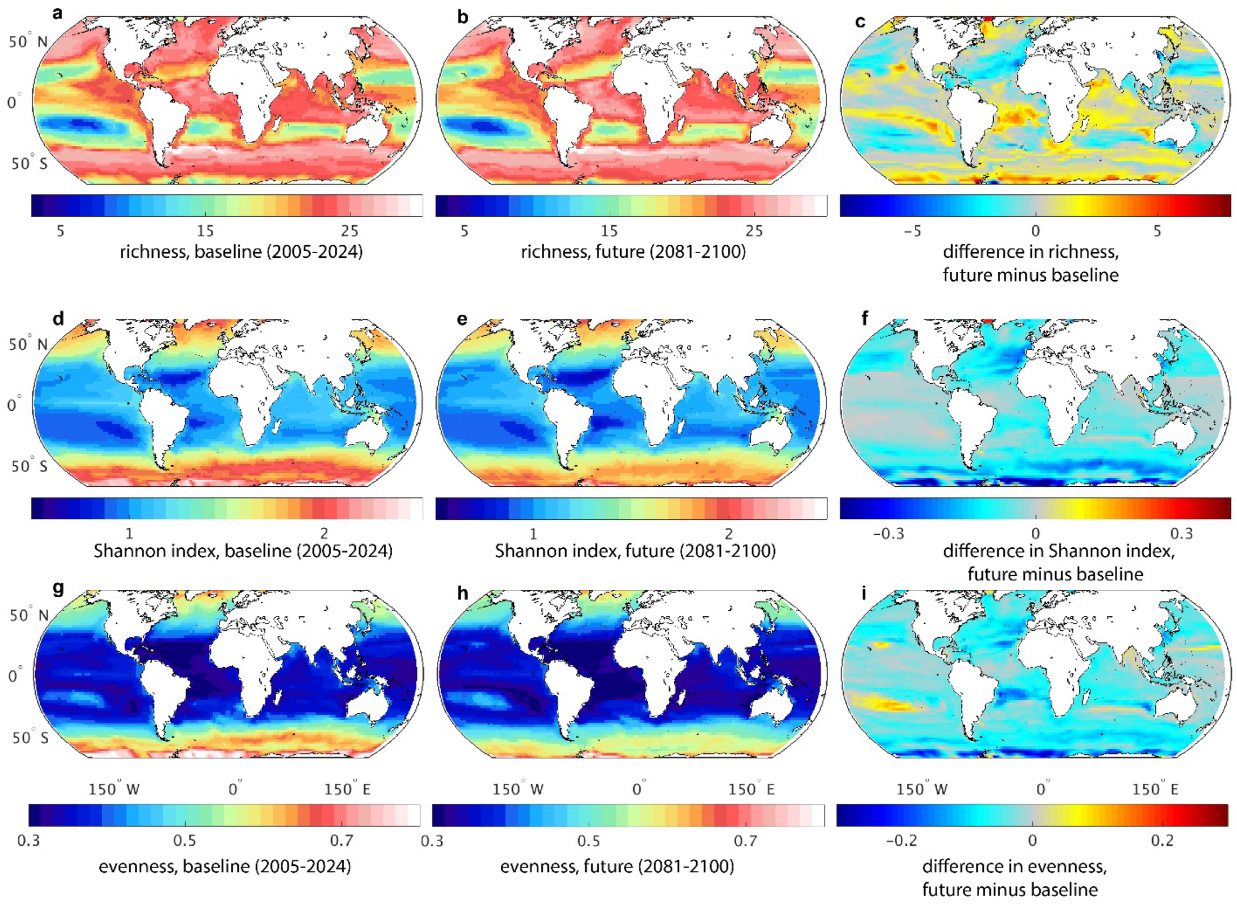

Supplementary Figure 5: Phytoplankton richness (a-c), Shannon index (d-f), and evenness of phytoplankton population (g-i) for mean of baseline period (2005-2024) in left column, mean of end of century period (2081-2100) in centre column, and difference between the end of century and baseline periods in right column.
